# Giant congenital melanocytic nevus in an Afghan child

**DOI:** 10.1002/ccr3.5258

**Published:** 2022-01-09

**Authors:** Nahid Raufi, Arash Nemat

**Affiliations:** ^1^ Department of Dermatology Maiwand Hospital Kabul University of Medical Sciences Kabul Afghanistan; ^2^ Department of Dermatology Guangdong Provincial Dermatology Hospital Southern Medical University Guangzhou China; ^3^ Department of Microbiology Kabul University of Medical Sciences Kabul Afghanistan; ^4^ Department of Cardiology Nanfang Hospital Southern Medical University Guangzhou China

**Keywords:** child, congenital melanocytic nevus, cutaneous, diagnosis, malignant transformation

## Abstract

We report an 8‐year‐old Afghan female with giant congenital melanocytic nevus (GCMN) which covered the entire back. The GCMN extended to anterolateral parts of the trunk surrounded by multiple satellite melanocytic nevi.

## Graphical abstract

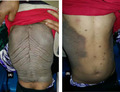

## 1 INTRODUCTION

Congenital melanocytic nevi (CMN) are cutaneous lesions characterized histologically by benign proliferations of melanocytes.^1^ GCMN in compare to CMN are deeper and extend more profound with irregular in margin, dark colored, and covered with coarse hair.^2^ Neurocutaneous melanoma is associated with GCMN which indicated bad prognosis of the disease.^3^ GCMN are often located on the trunk associated with hypertrichosis and satellite nevi. Several behavioral and emotional problems have been reported with GCMN. Recently numerous treatment options have been investigated for these patients, including surgical excision and laser therapy.^4^ Almost half of them develops by age 2 and 80% by age 7; thus, surgery at an early age is recommended. Removal of a GCMN requires a suitable donor site and several procedures to achieve the best esthetic outcome which in third‐world countries such as Afghanistan, repeat procedures with close follow‐up are typically not available.^5^

Here, we report a case of GCMN in a child in Afghanistan to highlight this pathology to the attention of clinicians. Although a recent report was published about an Afghan child with GCMN in Iran,^6^ to the best of our knowledge, GCMN has not been reported from inside Afghanistan.

## 2 CASE REPORT

An 8‐year‐old Afghan child who was born in Ghazni province of Afghanistan presented to our outpatient dermatology clinic with an extensive pigmented plaque over her body since birth. Her parents are first cousins with family history of spotted melanocytic nevi in her brother. Due to lack of healthcare access, she did not present until 8 years of age. When the lesions became symptomatic with dryness and pruritus, her parents sought evaluation and treatment. Her parents noted that she had asked many times about the dark spots and was distressed about them.

**FIGURE 1:**
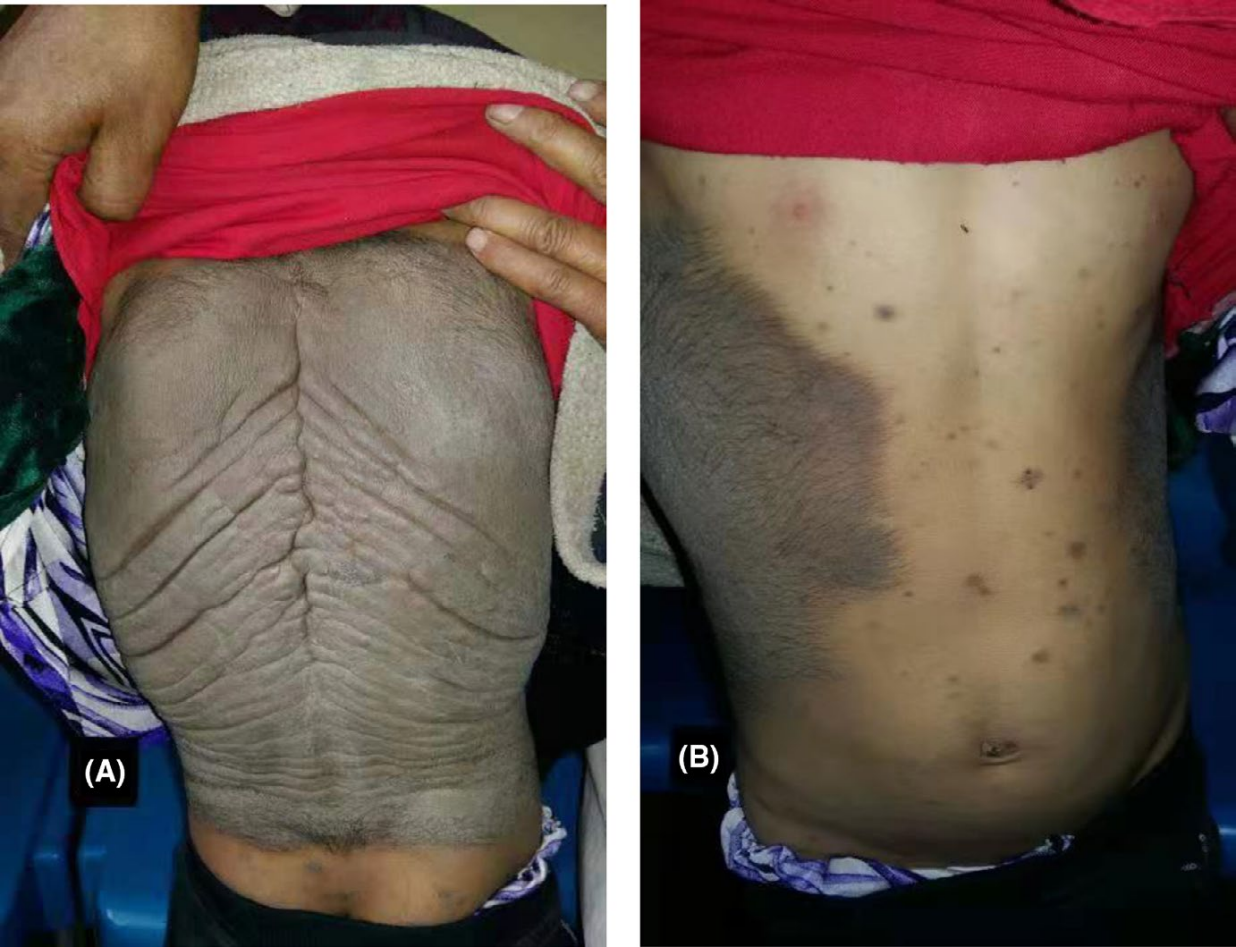
(A) Confluent dark brown plagues of the back (B) Extension to the anterolateral aspects of the trunk with hypertrichosis, with multiple satellite melanocytic nevi on the chest and abdomen

On examination, she had a dark brown melanocytic nevus with hairy rough surface that covered the entire back, reaching both anterolateral aspects of her trunk. (Shown in Figure 1).

Multiple satellite melanocytic nevi were also observed on her face, chest, and abdomen. No clinically atypical lesions were noted in the GCMN or satellite nevus. She did not have any neurological deficits with no radiological evidence for neural involvement. The patient had a depressed affect.

She was prescribed emollient and sunscreen. We referred the patient to the psychology department for evaluation and treatment of her depression. Unfortunately, the patient did not receive further investigations and treatment at dermatology department of our hospital. Because 1) most of the treatment options recommended in the early ages of the patients but our subject came to our department in her 8 years of age, 2) lack of necessary tools for diagnosis and treatment are serious problems in governmental hospitals of Afghanistan, and 3) follow‐up of the patients is rare in a country who is firing under conflict.

## 3 DISCUSSION

Giant congenital melanocytic nevus is a large hyperpigmented lesions commonly present since birth, these patches are trend to malignant transformation in the first 3–5 years of patients' life.^7^, ^8^ The GCMN is occurred 1 in 20,000 to 1 in 50,000 births.^2^ Due to its tendency to the malignant transformation, increased risk of neurological abnormalities, and cosmetic reasons the timely treatment is essential.^9^, ^10^ Laser therapy, skin grafting, the curettage of the nevus tissue in newborns, and the cultured epidermal autograft (CEA) are reported for GCMN treatment.^9^ The treatment options are mostly recommended in the early ages of the patients.^11^

To our knowledge, there are no published data from the Ministry of Public Health (MoPH) of Afghanistan on the prevalence of the CMN in the country. Less knowledge of the people, limited access of rural residents to health settings, and poverty let the patients to delay in their visit to the central clinics. Meanwhile, for those who present their illness, it is difficult to accept the clinicians' prescriptions particularly who need to cost and surgery.^12^ In the reality, the primary indication for such giant nevus, with high‐risk phenotypical features, large size and axial location, and multiple satellite lesions of the trunk, the serial excision of the lesion was recommended but unfortunately the patient was lost to follow‐up.

## CONFLICT OF INTEREST

The authors have no conflicts of interest to declare.

## AUTHOR CONTRIBUTIONS

The authors fulfill the ICMJE Criteria for Authorship and contributed equally. N.R. diagnosed the patient and wrote the case presentation. A.N prepared the introduction and discussion of the paper.

## ETHICAL APPROVAL

This study protocol was reviewed and approved Kabul University of Medical Sciences research committee, approval number was 21–0714 KUMS‐RC.

## CONSENT

The authors certify that they have obtained all appropriate patient consent forms. The parents of the child provided informed consent for publication of the case details and images. Institutional approval is not required for this case study. Written informed consent was obtained from mother of the patient to publish this report in accordance with the journal's patient consent policy.

## Data Availability

All data and images are available inside the paper.
